# Wheat homologs of yeast ATG6 function in autophagy and are implicated in powdery mildew immunity

**DOI:** 10.1186/s12870-015-0472-y

**Published:** 2015-04-01

**Authors:** Jieyu Yue, Hong Sun, Wei Zhang, Dan Pei, Yang He, Huazhong Wang

**Affiliations:** Tianjin Key Laboratory of Animal and Plant Resistance, School of Life Sciences, Tianjin Normal University, Tianjin, 300387 China

**Keywords:** Autophagy, ATG6, Common wheat (*Triticum aestivum* L.), Powdery mildew

## Abstract

**Background:**

Autophagy-related ATG6 proteins are pleiotropic proteins functioning in autophagy and the phosphatidylinositol 3-phosphate-signaling pathways. *Arabidopsis* ATG6 regulates normal plant growth, pollen development and germination, and plant responses to biotic/abiotic stresses. However, the ATG6 functions in wheat (*Triticum aestivum* L.), an important food crop, are lacking.

**Results:**

We identified three members, *TaATG6a*-*6c*, of the *ATG6* family from common wheat. *TaATG6a*, *6b* and *6c* were localized on homeologous chromosomes 3DL, 3BL and 3AL, respectively, of the allo-hexaploid wheat genome, and evidence was provided for their essential role in autophagy. The TaATG6a-GFP fusion protein was found in punctate pre-autophagosomal structures. The expression of each *TaATG6* gene restored the accumulation of autophagic bodies in *atg6*-mutant yeast. Additionally, *TaATG6* knockdown plants showed impaired constitutive and pathogen-induced autophagy and growth abnormalities under normal conditions. We also examined the expression patterns of wheat *ATG6*s for clues to their physiological roles, and found that their expression was induced by the fungus *Blumeria graminis* f. sp. *tritici* (*Bgt*), the causal agent of powdery mildew, and by abiotic stress factors. A role for TaATG6s in wheat immunity to powdery mildew was further implied when knockdowns of *TaATG6*s weakly compromised the broad-spectrum powdery mildew resistance gene *Pm21*-triggered resistance response and, conversely and significantly, enhanced the basal resistance of susceptible plants. In addition, leaf cell death was sometimes induced by growth-retarded small *Bgt* mycelia on susceptible *TaATG6* knockdown plants after a long period of interaction. Thus, we provide an important extension of the previous characterization of plant ATG6 genes in wheat, and observed a role for autophagy genes in wheat immune responses to fungal pathogens.

**Conclusions:**

Three wheat ATG6s were identified and shown to be essential for autophagy biogenesis. Wheat ATG6s are implicated in immunity to powdery mildew, playing a weak, positive role in the *Pm21*-triggered resistance response and a negative role in the basal resistance of susceptible plants.

**Electronic supplementary material:**

The online version of this article (doi:10.1186/s12870-015-0472-y) contains supplementary material, which is available to authorized users.

## Background

Unlike animals, plants are sessile and must overcome or endure variable, sometimes severe environmental conditions. For example, plants have evolved two main defense mechanisms, pathogen-associated molecular pattern (PAMP)-triggered immunity (PTI) and effecter-triggered immunity (ETI), to combat pathogen infections [1,2]. ETI is a much stronger reaction than PTI and is often accompanied by a localized hypersensitive response (HR), a type of programmed cell death (PCD), to capture the pathogen at the site of infection. Autophagy is an evolutionarily conserved, eukaryotic process by which organelles and cytosolic macromolecules are consumed in lysosomes (vacuoles in yeast and plants) for nutrient recycling [3-5]. Recent evidence from autophagy-defective *Arabidopsis* plants suggests an important role for autophagy in regulating plant immune responses [6,7]. This role, however, remains unclear since both positive and negative effects of autophagy on plant immunity have been described in different *Arabidopsis*-pathogen systems as well as in the same *Arabidopsis*-pathogen system [8,9]. Likewise, both pro-survival and pro-death roles of autophagy in the regulation of HR-PCD or pathogen-induced cell death have been reported [8-10]. Therefore, the mechanisms behind the relationship between autophagy and plant immunity need to be elucidated in detail when considering plant species other than *Arabidopsis* and more plant-pathogen systems.

During autophagy, double-membrane structures called autophagosomes facilitate the transport of cellular cargos into lysosomes for degradation [3-5]. To date, more than 30 autophagy-related proteins (ATGs) functioning in autophagy induction and regulation, the initiation and biogenesis of autophagosomes, and the fusion of autophagosomes with lysosomes have been identified in yeast and some orthologs identified in animals and plants [3-5]. In yeast, a Class III phosphatidylinositol 3-kinase (PI3K) complex resides in the pre-autophagosomal structure/phagophore assembly site (PAS) where it catalyzes phosphatidylinositol 3-phosphate (PI3P) synthesis and recruits PI3P-binding proteins, especially the ATG18-ATG2 complex, for the initiation of autophagic membranes [11-13]. Yeast ATG6/vacuolar protein sorting 30 (VPS30), the ortholog of mammalian Beclin 1, is the core component of the PI3K complex along with VPS34, VPS15 and ATG14 [14,15] and is essential for auophagy. Plant homologs of yeast ATG6 play similar essential roles in autophagy, for ATG6-deficient plants produce a reduced number of autolysosomes under autophagy-inducing conditions [16,17], and the expression of plant *ATG6* restores autophagy in *atg6*-mutant yeast [18].

ATG6-deficient plants, like mutants of other *atg* genes, show an enhanced sensitivity to nutrient starvation and other stress factors [17,19-22]. This enhanced sensitivity may result from impaired autophagy because autophagy is responsible for nutrient recycling and distribution under severe environmental conditions. However, the ATG6-associated PI3K complex is not autophagy-specific. Its phospholipid product, PI3P, is a well-known second messenger involved in receptor signaling and protein sorting-related vesicle trafficking events [23,24]. Homozygous plant mutants of *atg6*, *vps34* or *vps15* cannot be obtained because of the requirement of PI3K in pollen development, germination and pollen tube growth [25-27]. Such effects may be attributed to the PI3P-, or its derivative PI(3,5)P2-, signaling pathways rather than to autophagy because homozygous mutants of the autophagy-specific *atg* genes can be routinely obtained by selfing heterozygous individuals. ATG6-deficient plants also show more severe growth abnormalities under normal conditions than mutants of the autophagy-specific *atg* genes, although they have similarly reduced autophagy levels [17,22,25,28]. ATG6, like autophagy-specific factors, is implicated in plant immune responses [16,17]. Yet the pleiotropy of ATG6 makes it difficult to interpret whether autophagy, the PI3P-signaling pathways, or their interplay underlines the roles of ATG6 in plant immunity. Well-established roles of ATG6, other than autophagy, also include its involvement in the vacuolar protein sorting pathway of yeast and in tumor suppression through interactions with the anti-apoptotic Bcl-2-like proteins in mammals [14,29].

So far, few studies on the mechanisms and physiological functions of autophagy and other ATG6-associated pathways have been reported in crop plants, and fewer still in common wheat (*Triticum aestivum* L.), which is an important food crop worldwide. Here, we report the identification of three members of the wheat ATG6 family. The conserved function of wheat ATG6s in autophagy was shown using yeast functional complementation, subcellular localization of green fluorescent protein (GFP) fusions and the determination of autophagy levels in *ATG6* knockdown plants. A role for ATG6 in the wheat immune response to the causal fungus, *Blumeria graminis* f. sp. *tritici* (*Bgt*), of powdery mildew was established by *Bgt*-induced *ATG6* expression profiles and by altered powdery mildew symptoms on *ATG6* knockdown plants.

## Methods

### Plant materials and fungal strain

Wheat used in this study included isogenic lines 92R137/Yangmai 158^7^ and Yangmai 158, and isogenic lines Michigan Amber/Chancellor^8^ and Chancellor. 92R137/Yangmai 158^7^ carries the broad-spectrum powdery mildew resistance gene *Pm21*. Michigan Amber/Chancellor^8^ carries the isolate-specific powdery mildew resistance gene *Pm3f*. Yangmai 158 and Chancellor are susceptible to powdery mildew.

The prevalent Chinese *Bgt* isolate E09, which is avirulent to *Pm21* and *Pm3f*, was maintained on seedlings of the susceptible wheat cultivar Sumai 3 under spore-proof conditions.

### Plant growth conditions and treatments

All wheat plants were cultured hydroponically in one-fifth strength Hoagland’s solution under normal conditions of 22°C/18°C (day/night) and a photoperiod of 16 h light/8 h dark in controlled climate chambers. Two-leaf stage seedlings were subjected to different treatments. Pathogen inoculation was conducted by vigorously shaking off fresh *Bgt* conidia from diseased Sumai 3 plants onto experimental seedlings. For abiotic stress treatments, seedlings were transferred to the same one-fifth strength Hoagland’s solution containing 200 mM NaCl (high salinity) or 16% PEG6000 (drought). Additional abiotic stress treatments were nitrogen deprivation, and low temperature combined with darkness, in which seedlings were transferred to a 4°C refrigerator and kept in the dark. Leaves harvested at defined time points were frozen with liquid nitrogen and then stored at −80°C. These leaf samples were used for gene isolation and expression analyses.

### Isolation of wheat ATG6 genes and expression analyses

Total RNA was prepared from each harvested leaf sample using TRIzol (Invitrogen, Life Technologies, CA, USA) and subjected to RNase-free DNase I digestion and purification. First-strand cDNA was synthesized using oligo(dT) primers and the Quantscript RT Kit (Tiangen, Beijing, China).

The protein sequence of *Arabidopsis ATG6* (At3g61710) was used as the input to search for wheat expressed sequence tags (ESTs) in the dbEST division of GenBank by TBLASTN. Both 5ʹ- and 3ʹ-terminal ESTs were iteratively used as queries to search for new terminal ESTs to cover full-length open reading frames (ORFs). EST contigs were assembled in Vector NTI Advance 11.5, and their sequences were used to design primers (Additional file 2: Table S1) flanking full-length ORFs. ATG6 genes were amplified from first-strand cDNA using the high-fidelity Pfu DNA polymerase. Amplified products were A-tailed and then cloned into the vector pGEM®-T Easy (Promega, Madison, WI, USA) for sequencing. Sequence data were deposited in the GenBank database.

The expression patterns of wheat *ATG6*s responding to biotic and abiotic treatments were quantified by quantitative real-time PCR (qRT-PCR) with gene-specific primers (Additional file 2: Table S1). qRT-PCR was conducted in a 20 μl RealMasterMix (SYBR Green) mixture (Tiangen, Beijing, China) containing 1 μl first-strand cDNA as the template, and the reaction was performed on an IQ5 (BioRad, Hercules, CA, USA). Each cDNA sample was analyzed in triplicate. Relative expression levels were calculated using the 2^-ΔΔCT^ method [30] with the amplicon of the wheat β-tubulin gene as an internal control.

### Chromosomal localization of wheat *ATG6*s

Wheat nullisomic-tetrasomic lines (NTLs) were used to map ATG6 genes on specific chromosomes. DNA of each line was isolated and used as the template for PCR amplification with gene-specific primers (Additional file 2: Table S1). Amplified products were analyzed by 12% polyacrylamide gel electrophoresis. Chromosome localization was inferred based on the absence of amplified fragments from lines lacking specific chromosomes.

### Complementation test of yeast *atg6* mutant

The ORF of each wheat *ATG6* cDNA was amplified as a *Not*I- *Not*I fragment using the primers listed in Additional file 2: Table S1. Amplified products were digested and cloned into the yeast expression vector pFL61, in which the constitutive expression of the inserted gene was driven by the phosphoglycinecerate kinase (PGK) gene promoter. Constructed vectors were sequenced to confirm the correct cDNA insertion direction. Wild-type yeast strain BY4741 and a mutant strain, *atg6* (BY4741, *atg6Δ::kanMX MATa his3Δ1 leu2Δ0 met15Δ0 ura3Δ0*), were purchased from Open Biosystems (Thermo Scientific, Wilmington, DE, USA). Each constructed vector was introduced into yeast *atg6* cells according to the LiAc/SS-DNA/PEG TRAFO protocol [31]. Positive transformants were screened on SC-U medium. The yeast autophagy complementation test was performed as previously described [18]. The accumulation of autophagic bodies in vacuoles was observed under a differential interference contrast microscope (DM5000B, Leica).

### Subcellular localization of wheat ATG6s

The ORF of each wheat *ATG6* cDNA lacking a stop codon was amplified as an *Xho*I-*Spe*I fragment using primers listed in Additional file 2: Table S1. Amplified products were digested, and ligated upstream and in-frame with the ORF of *GFP* in the vector pA7-GFP, in which the *GFP* fusion construct’s expression was driven by two tandem 35S promoters. Prepared plasmids were introduced into onion epidermal cells by particle bombardment with PDS1000/He (BioRad, Hercules, CA, USA). GFP fluorescence was visualized by epifluorescence microscopy (DM5000B, Leica) 24 h after bombardment.

### Virus-induced gene silencing (VIGS)

A barley stripe mosaic virus (BSMV)-based VIGS method [32,33] was used to create gene knockdown plants. To maximize knockdown efficiency, three BSMV_γ_ vectors, each containing a 200–241 bp fragment from the upstream, middle, or downstream region of the *TaATG6a* coding sequence, were constructed, and *in vitro* transcribed RNA_γ_ molecules from these vectors were premixed for virus infection. To circumvent the potential functional redundancy among paralogs, we intended to produce simultaneous knockdowns of wheat *ATG6* members by choosing fragments from *TaATG6a* for the construction of BSMV_γ_ vectors with 94.1–99.5% sequence identities to the corresponding fragments of *TaATG6b* and *6c* and multiple nucleotide stretches longer than 23 bp that are 100% identical among the three *ATG6* members. The three fragments were prepared by PCR using the primers containing *Nhe*I sites listed in Additional file 2: Table S1. Each amplified fragment was digested by *Nhe*I and then inserted in the reverse orientation into the vector BSMV_γ_ to produce the vectors BSMV_γ:TaATG6as_-1, BSMV_γ:TaATG6as_-2 and BSMV_γ:TaATG6as_-3. Plasmids of these three BSMVγ derivatives, as well as BSMV_γ:GFP_ and BSMV_α_, were linearized by *Mlu*I, and BSMV_β_ was linearized by *Spe*I. Linearized vectors were *in vitro* transcribed to produce 5ʹ-capped infectious BSMV RNA molecules using the RiboMAX Large Scale RNA Production-T7 kit (Promega, Madison, WI, USA) with the addition of a cap analog (Promega, Madison, WI, USA) in the transcription mixture. RNA_γ:TaATG6as_-1, RNA_γ:TaATG6as_-2, and RNA_γ:TaATG6as_-3 were premixed in a 1:1:1 ratio to produce RNA_γ:TaATG6as_.

The second fully expanded leaves of wheat seedlings at the two-leaf stage were mechanically infected with a 1:1:1 mixture of RNA_α_, RNA_β_ and RNA_γ:TaATG6as_ (BSMV:ATG6 treatment) in 1× GKP buffer [33]. Seedlings infected with a 1:1:1 mixture of RNA_α_, RNA_β_ and RNA_γ:GFP_ (BSMV:GFP treatment) in 1× GKP buffer were used as controls. Three replications of the VIGS experiment were conducted on the two isogenic lines of 92R137/Yangmai 158^7^ and Yangmai 158. In each replication, a total of 80 seedlings per line were used for each of the BSMV:ATG6 and BSMV:GFP treatments. The BSMV-treated plants were maintained on one-fifth strength Hoagland’s solution under normal conditions. The success of the virus infection was judged by visual observations of mild chlorotic mosaic symptoms on the third and upper leaves of BSMV-treated plants. To confirm the knockdown efficiency, eight plants with virus symptoms were randomly selected from each of the two BSMV treatments, and their transcript levels of *TaATG6*s in the fourth leaves were determined using qRT-PCR as described above with a primer pair (Additional file 2: Table S1) designed to conserve regions in the three wheat *ATG6*s.

### Determination of autophagy level by LysoTracker Red staining

BSMV-treated plants at the four-leaf stage were inoculated with *Bgt* conidia as described above. The fourth leaves were excised at 0 and 7 days after inoculation (dai) with *Bgt*, infiltrated with 100 μM E-64d (Sigma, St. Louis, MO, USA) and then stained with 2 μM LysoTracker Red DND-99 (Invitrogen, Life Technologies, CA, USA) according to the procedure described previously [16]. Fluorescence signals were observed under a confocal microscope (ECLIPSE 90i, Nikon) using a 543-nm excitation laser and 565 to 615-nm band pass.

### Chlorophyll content determination

The fifth leaves of BSMV-treated plants at the five-leaf stage were excised, weighed and ground in 1 ml of 80% (v/v) acetone. The chlorophyll content was determined spectrophotometrically according to Patel and Dinesh-Kumar [17].

### Leaf senescence assay

Green sections were excised from the fourth leaves of BSMV-treated plants at the four-leaf stage and floated on deionized water at room temperature in the dark. Leaf sections were photographed every day after dark incubation. The experiment was repeated in triplicate using leaves of three or more plants in each experiment.

### Evaluation of powdery mildew resistance

To exclude the influence of early senescence/chlorosis on *Bgt* infections, only those BSMV:ATG6-treated plants without obvious symptoms of early senescence and chlorosis were selected for resistance evaluation. BSMV-treated plants at the six-leaf stage were inoculated with *Bgt* conidia as described above. For BSMV-treated Yangmai 158 plants, leaf discs with visible mildew colonies were gently harvested from different regions of the fourth leaves at 5 dai, immersed in 0.01% glycerol and subjected to an ultrasonic treatment for 1 min to disperse the conidia. The conidial production per mildew colony was counted by microscopic observation and expressed as the mean ± SD of at least 50 mildew colonies from five plants.

Fungal structures were stained by trypan blue [34] and visualized by light microscopy. For BSMV-treated 92R137/Yangmai 158^7^ (*Pm21*) plants, the number of successfully penetrating conidium with secondary hyphae on the fourth leaves was counted at 7 dai and expressed as a percentage of at least 100 interaction sites on individual plants. The number of secondary hypha associated with each successfully penetrating conidium was also counted and expressed as the mean ± SD of the observed successfully penetrating conidia on all of the investigated plants. For BSMV-treated Yangmai 158 plants, the number of mycelium with mature chains of conidia on the fourth leaves was counted at 5 dai and expressed as a percentage of at least 100 mycelia on the leaves of five plants.

### Statistical analysis

All experiments were repeated three times with similar results. Quantitative data were statistically analyzed using Student’s *t* test (P < 0.05) using the IBM SPSS19.0 software package.

## Results

### Isolation of *ATG6* homologs from wheat

Using the sequence of *Arabidopsis* ATG6 as a query, 44 putative wheat ATG6-encoding ESTs were mined from the dbEST division of GenBank and assembled into contigs. Primers covering the full-length ORFs of these contigs were designed and used for gene isolation by PCR from a leaf cDNA pool of the 92R137/Yangmai 158^7^ (*Pm21*) plants induced by *Bgt* for 48 h. Three genes, which encoded a typical ATG6 domain (Pfam PF04111) (Additional file 1: Figure S1A), were identified as *ATG6* homologs in wheat and designated as *TaATG6*a [GenBank: U49845], *6b* [GenBank: U49845] and *6c* [GenBank: U49845]. The ORF nucleotide sequences of *TaATG6a*, *6b* and *6c* share identities of 96.1–98.4% and encode peptide sequences of 504, 500, and 503 amino acids, respectively, with 98–99% similarity. In the eukaryotic ATG6 phylogenetic tree (Additional file 1: Figure S1B), plant ATG6s are separately grouped into monocot and dicot clades. TaATG6a-6c showed 90–93% sequence similarities to OsATG6a-6c from rice and 75% to AtATG6 from *Arabidopsis*. The ATG6 domain region is more conserved across all plant ATG6s (80–89% similarity), whereas the N- and C-terminal flanking sequences show divergence between the monocot and dicot ATG6s (Additional file 1: Figure S1A).

**Figure 1:**
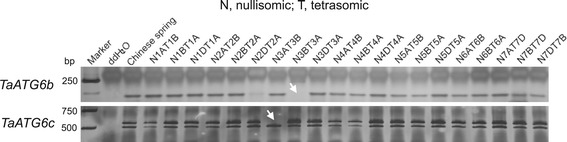
**Chromosomal localization of wheat autophagy-related ATG6 genes.** DNA of each wheat nullisomic-tetrasomic line was used as a template for PCR amplification with gene-specific primers. Amplified products were analyzed on 12% polyacrylamide gels. Chromosome localization was inferred based on the absence of amplified fragments (arrows) in lines lacking the specific chromosomes.

Two methods, gene-specific PCR amplification of wheat NTL DNAs and BLAST searches against the mapped wheat genomic sequences (genome survey sequences and the completed reference sequence of chromosome 3B) [35], were performed to determine the chromosomal localizations of wheat *ATG6* members. *TaATG6b* and *6c* were localized on chromosomes 3BL and 3AL, respectively, by both methods (Figure 1). *TaATG6a* was localized on chromosome 3DL based only on the results of BLAST searches.

**Figure 2:**
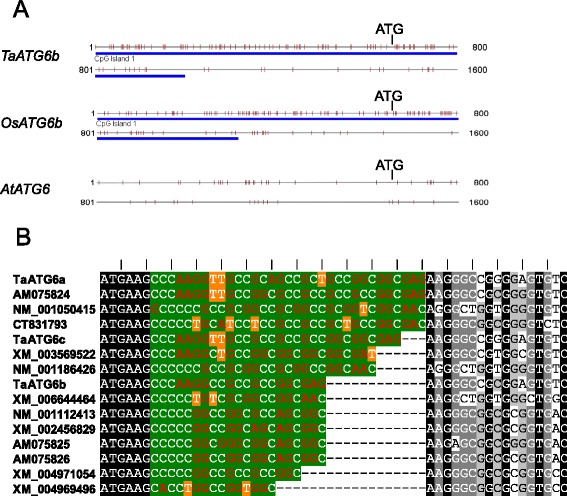
**Characterization of wheat autophagy-related** ***ATG6*** **sequences. (A)** The genomic sequences of monocot *ATG6*s contain a CpG island covering the start codon. The CpG island was predicted using the web tool CpG Island Searcher (http://cpgislands.usc.edu/) with a CpG island standard of longer than 500 bp in length, a minimum G + C content of 55% and a minimum 0.65 ratio of the observed versus the expected presence of CpG dinucleotides. Gray, horizontal lines represent the 1600-bp start codon-covering genomic sequences of *TaATG6b* from wheat, *OsATG6b* (Os03g0644000) from rice and *AtATG6* (At3g61710) from *Arabidopsis*. Short vertical red lines represent CpG dinucleotides. Blue horizontal thick lines represent the ranges of predicted CpG islands. **(B)** Short GC-rich stretch just downstream of the start codon in monocot *ATG6* sequences. Sequences shown are the 5ʹ-terminal cDNA sequences beginning with the start codons of monocot *ATG6*s, including *TaATG6a*, *6b* and *6c* from wheat, AM075824 from *Hordeum vulgare*, NM_001050415, CT831793 and NM_001186426 from rice, XM_003569522 from *Brachypodium distachyon*, XM_006644464 from *Oryza brachyantha*, NM_001112413 from *Zea may*s, XM_002456829 and AM075825 from *Sorghum bicolor*, AM075826 from *Saccharum officinarum*, and XM_004971054 and XM_004969496 from *Setaria italic*. The GC-rich stretches (green background) are not properly aligned but the flanking sequences are aligned. The reading frames from ATG are indicated by the upper vertical lines.

We also obtained the respective genomic sequences of *TaATG6a*, *6b* and *6c* by BLAST searches against wheat genomic sequences. Similar nine-intron structures of the *TaATG6a*-*6c* ORF sequences were revealed when comparing genomic sequences with the corresponding cDNA sequences (Additional file 1: Figure S2). However, *TaATG6b* has a much longer intron 3 than *TaATG6a* and *6c*. We compared the three genomic sequences and found a 900-bp insertion only in the intron 3 of *TaATG6b*, which is bordered by 19-bp direct short sequence repeats (TAGACTTAAATCATACTCC) (Additional file 1: Figure S2). As it encodes a truncated protein similar to RNA-directed DNA polymerases and polyproteins of mobile elements and because no long terminal repeats (LTR) were found around it, this inserted sequence most likely resulted from a transposition event of a non-LTR retrotransposon. A CpG island of 999 bp, covering the start codon, was detected in the *TaATG6b* genomic sequence, which has a 60.1% G + C content and a ratio of 1.07 of the observed versus expected presence of CpG dinucleotides (Figure 2A). This CpG island exists in other monocot *ATG6*s as well, including *OsATG6b* from rice, but not in dicot *ATG6*s, such as *AtATG6* from *Arabidopsis* (Figure 2A). Within the CpG island, there is a short GC-rich stretch just downstream of the start codon, and its length varies among paralogs as well as among homologs (Figure 2B). Only insertions or deletions that involve multiples of three bases are retained in the GC-rich stretch of monocot *ATG6*s because they do not result in translational frame shifts. Amino acids encoded in the GC-rich stretch are predominantly glycines and alanines.

### Wheat ATG6s function in autophagy

**Figure 3:**
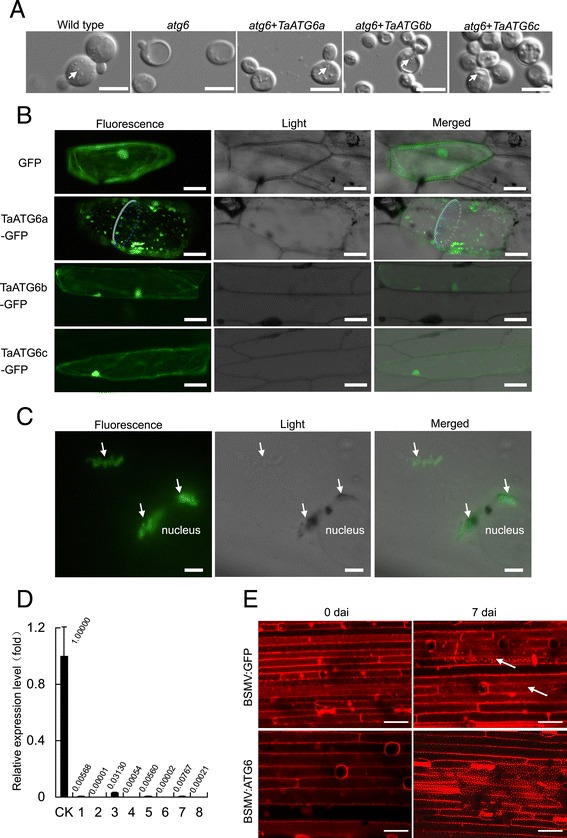
**Autophagy-related ATG6 genes function in wheat autophagy. (A)** Functional complementation of *atg6*-mutant yeast cells by wheat *ATG6*s. The autophagic bodies accumulated within vacuoles are indicated by arrows. Scale bars represent 5 μm. **(B)** Subcellular localization of GFP-TaATG6 fusion structures in onion epidermal cells. Scale bars represent 50 μm. **(C)** Localization of GFP-TaATG6a in cytoplasmic and perinuclear membrane-like structures in onion epidermal cells. Florescence spots localized in cytoplasmic and perinuclear membrane-like structures are indicated by arrows. Scale bars represent 10 μm. **(D)** Relative transcript levels of wheat *ATG6*s in leaves of barley stripe mosaic virus (BSMV)-treated plants. The fourth leaves were sampled from BSMV-treated 92R137/Yangmai 158^7^ (*Pm21*) plants under normal conditions and used in the quantification of *TaATG6* transcripts by qRT-PCR with a primer pair designed based on identical regions of *TaATG6a*, *6b*, and *6c*. Amplification of the wheat β-tubulin gene served as an internal control. CK is from BSMV:GFP-treated control plants and Arabic numbers indicate individual BSMV:ATG6-treated plants. **(E)** Constitutive and *Blumeria graminis* f. sp. *tritici* (*Bgt*)-induced autophagy are impaired in BSMV:ATG6-treated 92R137/Yangmai 158^7^ (*Pm21*) plants. The fourth leaves were excised from BSMV-treated plants at 0 and 7 days after inoculation (dai) with *Bgt* conidia, infiltrated with E-64d and then stained with LysoTracker Red. LysoTracker Red-stained punctate autolysosome-like structures are indicated by arrows. The results were reproduced from three independent experiments using three or more plants in each experiment. Scale bars represent 5 μm.

Human and *Arabidopsis* ATG6 can rescue the autophagy activity of *atg6*-mutant yeast [16,18,36]. To determine if wheat ATG6s are functional homologs of yeast ATG6, yeast expression vectors were constructed for each *TaATG6* and transformed into *atg6*-mutant yeast cells. Autophagic bodies in vacuoles were monitored as an indicator of autophagy activity. After 5 h of nutrient starvation and treatment with the protease inhibitor phenylmethylsulfonyl fluoride, the wild-type yeast cells accumulated numerous autophagic bodies in vacuoles, which were rarely observed in the *atg6*-mutant yeast cells (Figure 3A). Cells of *atg6*-mutant yeast transformed with *TaATG6a*, *6b* or *6c* partially restored the accumulation of autophagic bodies (Figure 3A).

Punctate structures of ATG6-GFP fluorescence, considered as PAS for autophagosome initiation, have been observed in cells of yeast and *Arabidopsis* [18,37]. Here, *GFP* fusion constructs of wheat *ATG6*s were introduced into onion epidermal cells by particle bombardment. Punctate florescence signals were clearly observed in the cytoplasm of cells expressing TaATG6a-GFP and seemed not to occur in the central vacuole (Figure 3B). Fluorescent spots of TaATG6a-GFP were sometimes clustered at cytoplasmic and perinuclear membrane-like structures resembling the trans-Golgi network and endoplasmic reticulum (Figure 3C). Localizations of ATG6s to endosomes, the trans-Golgi network, endoplasmic reticulum, mitochondria, cytoplasm, and nucleus have been reported in yeast and human [37-40]. Unexpectedly, TaATG6b- and TaATG6c-GFP florescence showed diffused distributions in the cytoplasm and nuclei, which were almost indistinguishable from those in cells expressing GFP alone (Figure 3B). Therefore, additional signals may be required to trigger the translocation of TaATG6b and 6c to PAS for their role in autophagy initiation.

To determine if an ATG6 deficiency can lead to autophagy impairment in wheat, BSMV:ATG6-treated *TaATG6* knockdown plants were generated by the VIGS method and their autophagy levels were compared with BSMV:GFP-treated control plants. To obtain the maximum knockdown efficiency, BSMV:ATG6 was designed to contain a mix of three BSMV RNA_γ_ molecules, each containing an inserted antisense fragment corresponding to the upstream, middle, and downstream regions of the *TaATG6a* coding sequence. Taking into consideration the potential functional redundancy among paralogs in polyploid wheat, BSMV:ATG6 was also designed to knock down all three wheat *ATG6* members by inserting three fragments in the RNA_γ_ molecules that are highly conserved (more than 94.1% identical) among the three genes. As determined by qRT-PCR using a primer pair designed based on identical regions of the three *TaATG6*s, the total constitutive transcript levels of *TaATG6a*-*6c* were greatly reduced in the BSMV:ATG6-treated plants compared with the transcript levels in BSMV:GFP-treated control plants (Figure 3D). This high knockdown efficiency was achieved in all eight BSMV:ATG6-treated plants investigated, which confirmed the effectiveness of our VIGS design. We then inoculated BSMV-treated plants with *Bgt* conidia and determined their constitutive autophagy activity at 0 dai and *Bgt*-induced autophagy activity at 7 dai using the LysoTracker Red staining method. LysoTracker Red is a dye that stains acidic organelles, such as lysosomes and endosomes, as well as autophagosomes/autolysosomes (vacuoles and small lytic organelles). Punctate structures labeled by this dye are generally considered indicative of plant autophagy activity [16,41-44]. Before LysoTracker Red staining, leaf samples were pre-infiltrated with the cysteine protease inhibitor E-64d to enhance the accumulation of autophagic bodies inside autolysosomes [16,45,46]. For the BSMV:GFP-treated control plants of the resistant line 92R137/Yangmai 158^7^ (*Pm21*), low levels of a LysoTracker Red-stained punctate pattern were observed at 0 dai, and enhanced levels were observed at 7 dai in *Bgt*-challenged leaf epidermal cells (Figure 3E). These *Bgt*-enhanced levels implied that the TaATG6s-regulating autophagy process was induced and implicated in the *Pm21*-triggered immune response to powdery mildew. However, because of the knocking down of *TaATG6*s, the LysoTracker Red-stained punctate structures were not visible in leaf samples of BSMV:ATG6-treated plants regardless of *Bgt* challenge (Figure 3E). This result of impaired autophagy in *TaATG6* knockdown plants, together with the complementation of yeast autophagy-defective cells with each *TaATG6* and the observation of PAS localization of TaATG6a-GFP, demonstrates the essential role of TaATG6s in wheat autophagy.

### Wheat *ATG6* knockdown plants exhibited growth abnormalities

**Figure 4:**
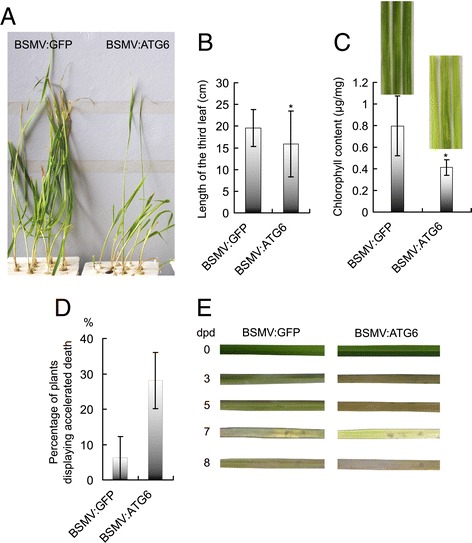
**Wheat autophagy-related** ***ATG6*** **knockdown plants display growth abnormalities.** All barley stripe mosaic virus (BSMV)-treated plants were cultured hydroponically in one-fifth strength Hoagland’s solution and kept under normal conditions of 22°C/18°C (day/night) and a photoperiod of 16 h light/8 h dark in controlled climate chambers. **(A)** The growth morphology of BSMV-treated Yangmai 158 plants. Photographs were taken at the three-leaf stage. The second leaves, which had been mechanically infected with BSMV, were generally withered by this stage and thus were excised before photographing. **(B)** Leaf length of BSMV-treated Yangmai 158 plants. The lengths of the third leaves were measured at the three-leaf stage. Values represent the mean ± SD (standard deviation) of data measured from 30 individual plants. Asterisks indicate a significant difference (P < 0.05, Student’s *t* test) between the BSMV:ATG6-treated plants and BSMV:GFP-treated control plants. **(C)** Chlorophyll content of BSMV-treated Yangmai 158 plants. The chlorophyll content of the fifth leaves was determined at the five-leaf stage. Values represent the mean ± SD of data determined from five individual plants. Asterisks indicate a significant difference (P < 0.05) between the BSMV:ATG6-treated plants and BSMV:GFP-treated control plants. **(D)** Percentage of BSMV-treated Yangmai 158 plants displaying accelerated death before the five-leaf stage. Values represent the mean percentage ± SEM (standard error of the mean) of at least 30 plants. **(E)** Detached leaves from BSMV:ATG6-treated plants display accelerated dark-induced senescence. Green sections of the fourth leaves were excised from BSMV-treated Yangmai 158 plants at the four-leaf stage and were floated on deionized water at room temperature in the dark. Leaf sections were photographed at the indicated days post-detachment (dpd). The results are reproduced from three independent experiments using the leaves of three or more plants in each experiment.

BSMV:ATG6-treated plants, when compared with control plants, showed varying degrees of growth defects, including stunted growth and chlorosis under normal conditions (Figure 4A–C). Some were thin and weak, and a higher percentage of BSMV:ATG6-treated plants shifted into early senescence and accelerated death before the five-leaf stage than of the controls (Figure 4D). To further monitor senescence, green leaf sections from plants at the same developmental stage were excised and kept floating on water in the dark to induce senescence. Leaf sections from BSMV:ATG6-treated plants began senescing within 3 d post-detachment, as indicated by yellowing (Figure 4E). This was significantly earlier than leaf sections from control plants, which began senescing by 7 d post-detachment. Thus, TaATG6s are essential for normal plant growth and to prevent premature senescence in wheat.

### Expression of wheat *ATG6*s in response to abiotic stress factors and fungal infection

**Figure 5:**
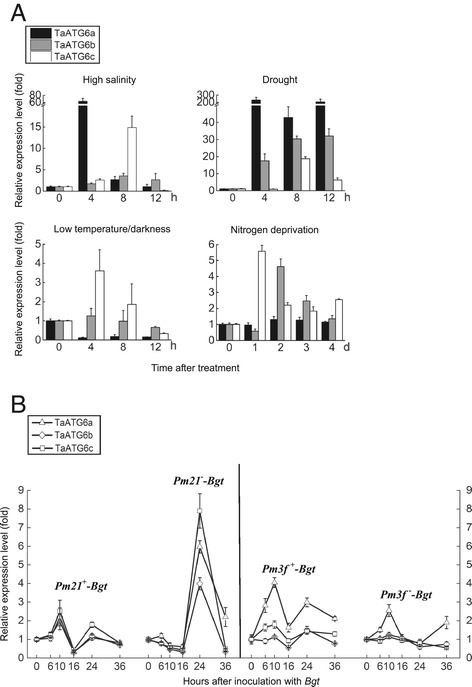
**Expression patterns of wheat autophagy-related ATG6 genes.** Two-leaf stage seedlings were subjected to different treatments. Samples were collected at the indicated time points after the initiation of treatment. Total RNA was extracted and qRT-PCR was performed with gene-specific primers. The relative expression levels were normalized by the wheat β-tubulin gene and relative to the control value measured at 0 h. Data represent the mean of three independent experiments ± SD. **(A)** Expression of wheat *ATG6*s in response to abiotic stress factors. Yangmai 158 seedlings were transferred to the one-fifth strength Hoagland’s solution containing 200 mM NaCl (high salinity) or 16% PEG6000 (drought). Additional seedling treatments were nitrogen deprivation and exposure to 4°C in the dark (low temperature/darkness). **(B)** Expression of wheat *ATG6*s in response to *Blumeria graminis* f. sp. *tritici* (*Bgt*) infection. Pathogen inoculations were conducted by vigorously shaking fresh *Bgt* conidia from diseased plants onto seedlings of the isogenic lines 92R137/Yangmai 158^7^ (*Pm21* ^+^) and Yangmai 158 (*Pm21* ^−^), and the isogenic lines Michigan Amber/Chancellor^8^ (*Pm3f* ^+^) and Chancellor (*Pm3f* ^−^).

Yangmai 158 plants were studied using qRT-PCR to determine the effects of abiotic stress factors on the expression of wheat *ATG6*s. The expression levels of *TaATG6b* and *6c* were upregulated by high salinity, drought, low temperature/darkness and nitrogen deprivation (Figure 5A). The expression of *TaATG6a* was also upregulated by high salinity, drought and nitrogen deprivation, but downregulated by low temperature/darkness (Figure 5A). Among the four investigated abiotic stress factors, wheat *ATG6*s were most sensitive to the two osmotic stress factors, high salinity and drought, with up to 63-fold (*TaATG6a* responding to high salinity) and 230-fold (*TaATG6a* responding to drought) increases in the accumulation of transcripts. These upregulated expression modes indicate that TaATG6s are closely related to the wheat’s responses to abiotic stress factors.

Two powdery mildew resistant lines, 92R137/Yangmai 158^7^ (*Pm21*) and Michigan Amber/Chancellor^8^ (*Pm3f*), and their respective isogenic susceptible lines were included in the expression analysis, using qRT-PCR, of wheat *ATG6*s responding to *Bgt* infection. In the broad-spectrum resistance gene *Pm21*- and the isolate-specific resistance gene *Pm3f*-triggered resistance responses, the expression of wheat *ATG6*s was upregulated by *Bgt*, producing bimodal profiles over a time interval of 0 to 36 h after inoculation (hai) with *Bgt* (Figure 5B). The two peaks of transcript accumulation occurred at 10 and 24 hai. Upon *Bgt* challenge, *Pm21* activated the three *ATG6*s to about the same extent, while *Pm3f* activated *TaATG6a* to a greater extent than *TaATG6b* and *6c*. In the susceptible response of Yangmai 158 to *Bgt*, however, a single instance of upregulated expression within 16 to 24 hai was detected for the three *ATG6*s (Figure 5B). This corresponded to, but was higher than, the second instance of upregulated expression detected in the two resistance responses. In the susceptible response of Chancellor to *Bgt*, only one member, *TaATG6a*, showed a *Bgt*-induced expression profile (Figure 5B). These *Bgt*-upregulated expression patterns implied that TaATG6s are involved in the wheat immune response to powdery mildew.

### Wheat ATG6s play a weak, positive role in the *Pm21*-triggered resistance response to *Bgt*

**Figure 6:**
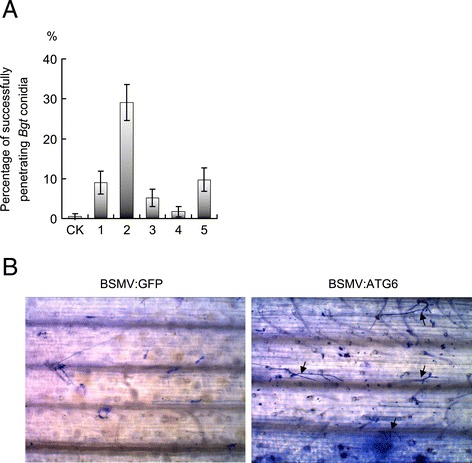
**Knocking down wheat autophagy-related ATG6 genes weakly compromises the broad-spectrum resistance gene** ***Pm21*** **-triggered resistance response to** ***Blumeria graminis*** **f. sp.** ***tritici*** **(** ***Bgt*** **).** Barley stripe mosaic virus (BSMV)-treated 92R137/Yangmai 158^7^ (*Pm21*) plants of the six-leaf stage were heavily inoculated with *Bgt* conidia. Fungal structures were stained by trypan blue and visualized by microscopy. **(A)** *Bgt* penetration frequency. The number of successfully penetrating conidium with secondary hyphae on the fourth leaves was counted at 7 days after inoculation (dai) with *Bgt* conidia. Values represent the mean percentage of successfully penetrating *Bgt* conidia ± SEM calculated from at least 100 interaction sites. CK is from BSMV:GFP-treated 92R137/Yangmai 158^7^ (*Pm21*) control plants. Arabic numbers indicate individual BSMV:ATG6-treated 92R137/Yangmai 158^7^ (*Pm21*) plants. **(B)** Representative images of *Bgt* infection on the fourth leaves of BSMV-treated 92R137/Yangmai 158^7^ (*Pm21*) plants at 7 dai. Successfully penetrating conidia with secondary hyphae are indicated by arrows.

We used *TaATG6* knockdown plants to test if TaATG6s have any effect on *Bgt* infection. To exclude the influence of early senescence/chlorosis on *Bgt* infection, only those knockdown plants that did not show obvious early senescence and chlorosis were selected for resistance evaluation. BSMV-treated plants were heavily inoculated with *Bgt* conidia, and disease symptoms were determined when mildew colonies were apparently visible on susceptible plant leaves. The knocking down of *TaATG6*s did not result in visible mildew colonies on leaves of BSMV:ATG6-treated 92R137/Yangmai 158^7^ (*Pm21*) plants by 7 dai, which seemed to retain a resistant phenotype similar to BSMV:GFP-treated control plants. We then stained leaf samples with trypan blue and, under a microscope, counted the number of successfully penetrating *Bgt* conidium with secondary hyphae. As shown in Figure 6A and B, the percentage of successfully penetrating *Bgt* conidia (penetration frequency) was very low (0.5%) on leaves of the highly resistant BSMV:GFP-treated control plants, but significantly increased to 1.7–29.1% on leaves of different BSMV:ATG6-treated plants. Furthermore, successfully penetrating *Bgt* conidia on leaves of BSMV:ATG6-treated plants developed an average of 4.16 ± 2.91 prolonged secondary hyphae, whereas only one short secondary hypha was associated with successfully penetrating *Bgt* conidia on leaves of BSMV:GFP-treated control plants (Figure 6B). These results indicated that knocking down *TaATG6*s resulted in a compromised *Bgt* resistance on 92R137/Yangmai 158^7^ (*Pm21*) plants. Therefore, we propose that TaATG6s play a positive role in *Pm21*-triggered wheat resistance response to *Bgt* and that this positive role is weak since no large, sporulating mycelia were observed on *TaATG6* knockdown plants of 92R137/Yangmai 158^7^ (*Pm21*) by 7 dai. In *ATG6*, *VPS34*, *ATG3*, or *ATG7* knockdown tobacco plants and in *ATG6* knockdown or *atg5* mutant *Arabidopsis* plants, avirulent viral or bacterial pathogen-induced HR-PCD was unrestricted and spread into uninfected cells, suggesting that autophagy has a pro-survival function in the pathogen-induced cell death response [16,17,44,47]. In our study, however, such an unrestricted spread of cell death was not induced by the biotrophic fungal pathogen *Bgt* on *TaATG6* knockdown plants of 92R137/Yangmai 158^7^ (*Pm21*).

### Wheat ATG6s also play a negative role in the basal resistance of plants susceptible to *Bgt*

**Figure 7:**
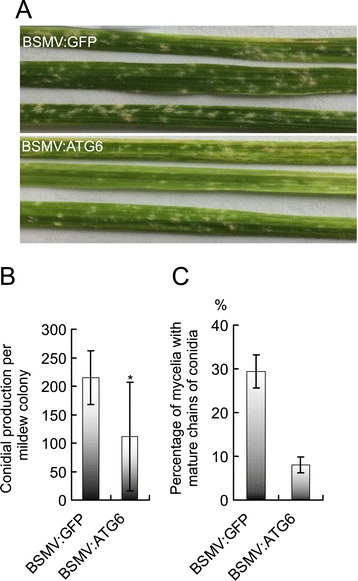
**Knocking down wheat autophagy-related ATG6 genes enhances the basal resistance of susceptible plants to** ***Blumeria graminis*** **f. sp.** ***tritici*** **(** ***Bgt*** **).** Barley stripe mosaic virus (BSMV)-treated Yangmai 158 plants of the six-leaf stage were heavily inoculated with *Bgt* conidia. **(A)** Representative images of mildew colonies on the fourth leaves of BSMV-treated Yangmai 158 plants. Images were taken at 5 days after inoculation (dai) with *Bgt* conidia. **(B)** Sporulation of *Bgt* colonies on BSMV-treated Yangmai 158 plants. Leaf discs with visible mildew colonies were gently harvested from different regions of the fourth leaves at 5 dai, immersed in 0.01% glycerol and subjected to ultrasonic treatment for 1 min to disperse the conidia. The conidial production per mildew colony was counted by microscopic observation. Values represent the mean conidial production per mildew colony ± SD of at least 50 mildew colonies from five plants. Asterisks indicate a significant difference (P < 0.05, Student’s *t* test) between the BSMV:ATG6-treated plants and BSMV:GFP-treated control plants of Yangmai 158. **(C)** Percentage of mycelia with mature chains of conidia on BSMV-treated Yangmai 158 plants. Fungal structures were stained by trypan blue and visualized by light microscopy. The number of mycelium with mature chains of conidia on the fourth leaves was counted at 5 dai. At least 100 mycelia from five individual plants were counted and values represent the mean percentage of mycelia with mature conidia ± SEM.

**Figure 8:**
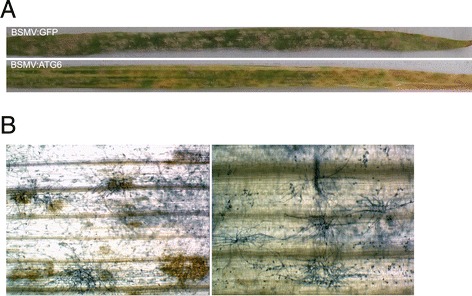
**Lesions and chlorosis are induced by** ***Blumeria graminis*** **f. sp.** ***tritici*** **(** ***Bgt*** **) on wheat autophagy-related** ***ATG6*** **knockdown susceptible plants.. (A)** Representative images of mildew colonies, leaf lesions and chlorosis on the sixth leaves of BSMV-treated Yangmai 158 plants at 10 days after inoculation (dai) with *Bgt* conidia. **(B)** Microscopic observation of growth-retarded small mycelia associated (left panel) or not associated (right panel) with leaf cell death on BSMV:ATG6-treated Yangmai 158 plants at 10 dai.

Although the BSMV:ATG6-treated plants and BSMV:GFP-treated control plants of Yangmai 158 were both colonized by *Bgt* at 5 dai, the sizes of visible mycelia and their sporulation (conidial production per mildew colony) were significantly reduced on both lower leaves (the fourth leaves, Figure 7A and B) and upper leaves (the sixth leaves, Figure 8A) of BSMV:ATG6-treated plants. *Bgt* mycelia were also examined under a microscope after trypan blue staining, and a larger portion of small mycelia and a significantly reduced percentage of mycelia with mature chains of conidia were observed on leaves of BSMV:ATG6-treated plants at 5 dai (Figure 7C). Clearly, a significantly enhanced resistance should be responsible for the retarded mycelial spread and sporulation on *TaATG6* knockdown plants. We thus proposed that TaATG6s play negative roles in the basal resistance of the susceptible line Yangmai 158 to powdery mildew. Moreover, growth-retarded small mycelia, after a prolonged infection time of 9–10 dai, induced lesions and chlorosis around lesions on the middle-to-tip leaf regions of BSMV:ATG6-treated Yangmai 158 plants (Figure 8A, left panel of 8B). The exhibition of enhanced resistance preceded the occurrence of leaf lesions and not all small mycelia were finally associated with leaf cell death (Figure 8B), although leaf cell death may further impair the biotrophic lifestyle of *Bgt*.

## Discussion

As a key component of the Class III PI3K kinase complex, ATG6 is essential for autophagy and the PI3P-signaling pathways. Previously, plant ATG6 orthologs had been identified in *Nicotiana benthamiana*, *Arabidopsis* and rice, and their involvement in autophagy had been demonstrated [16,17,48]. Here, we characterized three wheat ATG6 genes, *TaATG6a*, *6b* and *6c*, which were localized on the homeologous chromosomes 3DL, 3BL and 3AL, respectively, of the allo-hexaploid wheat genome (AABBDD). Heterologous expression of *TaATG6a*, *6b*, or *6c* can restore autophagy in *atg6*-mutant yeast cells. The TaATG6a-GFP fusion protein was located in cytoplasmic punctate PAS structures. Constitutive and pathogen-induced autophagy processes were impaired in *TaATG6* knockdown plants. Collectively, these results support the essential role of TaATG6s in wheat autophagy biogenesis. Consistent with *Arabidopsis* plants deficient in the PI3K components ATG6 or VPS34 [17,22,49], *TaATG6* knockdown wheat plants exhibit abnormal growth phenotypes, such as dwarfing, chlorosis, and accelerated senescence/death, under normal conditions. However, the role of PI3K on normal plant growth may be beyond autophagy, as mutants of autophagy-specific *atg* generally do not show such extensive growth abnormalities under normal conditions [19-21,28,50].

Pathogen-induced expression patterns and positive roles of ATG6 in plant innate immunity have been reported in plant pathosystems, such as *N. benthamiana*-TMV [16] and *Arabidopsis*-*Pseudomonas syringae* [17]. Here, two transcript accumulation events, an early and a late one, of wheat *ATG6s* were induced in the *Pm21*- and *Pm3f*-triggered wheat resistance responses to *Bgt* within 0 to 36 hai. *Bgt*-induced bimodal expression profiles have also been characterized for other powdery mildew resistance-related genes in wheat [51,52]. Two pathogen-induced oxygen burst processes reported previously also match the two time points [53]. The early wheat *ATG6* transcript accumulation event occurred within 0 to 10 hai, which is when *Bgt* appressorium germ tubes contact and attempt to invade wheat epidermal cells. This transcript accumulation may be pivotal for the *Pm21*-triggered resistance response because it was not detected in the isogenic susceptible plants of Yangmai 158. Although knocking down *TaATG6*s did not result in a striking change in the *Pm21*-triggered resistance phenotype, an increased penetration frequency of inoculated *Bgt* conidia was observed by microscopy on knockdown plants of 92R137/Yangmai 158^7^ (*Pm21*). Since successfully penetrating conidia on knockdown plants were inevitably arrested before extending into large mycelia, we hypothesize that TaATG6s play only a weak positive role, probably in the early phase of *Bgt* invasion, in the *Pm21*-triggered resistance response. Recent studies on autophagy-specific *atg* gene mutants support the involvement of autophagy in plant immune responses [6,8]. The *Bgt*-enhanced occurrence of LysoTracker Red-stained autolysosome-like structures in control plants of 92R137/Yangmai 158^7^ (*Pm21*), which “disappeared” in *TaATG6* knockdown plants, also links autophagy to ATG6 during the wheat immune response to *Bgt*. Positive roles of autophagy in plant resistance responses have been reported [16,17,43,44,54]. Similar to our finding of a weak, positive role of TaATG6s in the *Pm21*-triggered resistance response to *Bgt*, *Arabidopsis atg7* and *atg9* mutants are compromised in the early restriction of avirulent *P. syringae* growth, but retain the capacity to induce other defense responses [42].

Contrary to their positive roles in the *Pm21*-triggered resistance response, TaATG6s had negative roles in the basal resistance of the susceptible line Yangmai 158. Knocking down *TaATG6*s enhanced the resistance of Yangmai 158 plants to *Bgt* mycelial extension and sporulation. Our expression data showed that, compared with their performances in resistance plants of 92R137/Yangmai 158^7^ (*Pm21*), TaATG6s were absent for the first response to *Bgt*, which occurred within 0–10 hai, but were intensely involved in the second response to *Bgt*, which occurred after 16 hai, in the susceptible plants of Yangmai 158. The large quantity of successfully penetrating *Bgt* conidia on susceptible plants may induce the highly upregulated expression of *TaATG6*s after 16 hai. The retarded mycelial spread and sporulation on *TaATG6* knockdown susceptible plants suggested that, in wild-type susceptible plants, the ATG6 recruitment after 16 hai might be beneficial for the further development of *Bgt* mycelium. Thus, high levels of TaATG6s, and certain TaATG6-regulated pathways, in susceptible host cells might be hijacked by *Bgt* after the penetration stage to facilitate its pathogenicity, leading them to function negatively in the basal resistance of susceptible plants. The autophagy process in host cells is a potential target because of its function in the remobilization of cellular nutrients, which may be exploited by *Bgt* to meet the nutrient supply for fungus proliferation. Negative roles of autophagy in plant basal resistance have been reported in the susceptible responses of *Arabidopsis* to the powdery mildew fungus *Golovinomyces cichoracearum* [55] and to the bacterial pathogen *P. syringae* [44,54]. However, we failed to observe autophagy activity in *Bgt*-infected cells of wild-type Yangmai 158 plants using the LysoTracker Red staining method, probably because of the very low induction level (data not shown). Considering this and the pleiotropy of ATG6, we cannot exclude other ATG6- or PI3P-regulated pathways, or their interplay with autophagy, in underpinning the negative role of ATG6 in the basal resistance of susceptible wheat plants to powdery mildew.

Cell death was induced by growth-retarded small mycelia of *Bgt* on the leaves of *TaATG6* knockdown plants of Yangmai 158. However, cell death does not seem to be the reason for the enhanced resistance because it occurred after the exhibition of enhanced resistance and was not induced by all small mycelia. Similarly, powdery mildew induced lesions, but they were not necessarily coupled with the enhanced resistance of susceptible *Arabidopsis atg2* mutants [55]. The enhanced resistance of *Arabidopsis pmr* mutants to the powdery mildew pathogen *Erysiphe cichoracearum* and autophagy-defective mutants to *P. syringae* is also not associated with cell death [54,56]. Since ATG2 is autophagy-specific, the powdery mildew-induced cell death of susceptible *Arabidopsis atg2* mutants indicates a pro-survival role of autophagy in this compatible interaction [55]. However, in view of the weak *Bgt*-induced autophagy level and the pleiotropy of ATG6, whether autophagy alone or combined with other ATG6-regulated pathways underlies the suppression function of TaATG6s on *Bgt*-induced cell death of susceptible wheat plants needs to be determined.

## Conclusions

In this study we provide evidence from yeast mutant complementation, subcellular localization of GFP fusions, and detection of autophagy levels in knockdown plants to show that the three identified wheat ATG6s are essential for autophagy biogenesis. *Bgt*-induced expression patterns, and the altered symptoms of powdery mildew on knockdown plants, suggest that TaATG6s are implicated in wheat immunity to *Bgt*, playing a weak, positive role in the *Pm21*-triggered resistance response and a negative role in the basal resistance of the susceptible line Yangmai158. These findings lay the foundations for a better understanding of autophagy mechanisms in crop plants and the role of ATG6 in wheat immunity.

## Additional files

Additional file 1: Figure S1. Sequence comparison and phylogenetic relationship of eukaryotic autophagy-related ATG6 proteins. (A) Sequence comparison between wheat ATG6 proteins and their homologs. The predicted amino acid sequences of wheat TaATG6a, 6b and 6c were aligned with homologous ATG6s from *Arabidopsis* (AtATG6, AAK62668), yeast (ScATG6, Q02948) and human (HsATG6, Q14457). The alignment was generated in ClustalX 2.1 and viewed in the GeneDoc 2.7 program. Numbers on the right indicate amino acid residue positions. Similarity was coded as follows: 100%, black; 80 to 100%, dark gray; 60 to 80%, light gray; and < 60%, white. The ATG6 domain (Pfam PF04111) region is under the straight lines. (B) Phylogenetic relationship of plant ATG6s. The phylogenetic tree was generated with MEGA 5 using the neighbor-joining method. The reliability of internal branches was assessed by bootstrapping, with 1000 bootstrap replicates, and the values are shown in percentages, with a branching cut-off at 50%. Wheat ATG6s are indicated by diamonds. Figure S2. Diagram of the exon-intron structures of wheat *ATG6* genomic ORF sequences. Exons (boxes) and introns (lines between boxes) are drawn to scale, except introns indicated by question marks, which represent there are gaps between available genomic sequences. Two vertical short lines in intron 3 of *TaATG6b* represent direct short sequence repeats (TAGACTTAAATCATACTCC) delimiting the non-LTR retrotransposon (RT) sequence. Additional file 2: Table S1. List of primers used in this paper.

## Abbreviations

ATG: Autophagy-related gene
BSMV: Barley stripe mosaic virus
GFP: Green fluorescent protein
PAS: Pre-autophagosomal structure/phagophore assembly site
PI3K: Phosphatidylinositol 3-kinase
VIGS: Virus-induced gene silencing
