# Supplementary material for: Wheat homologs of yeast ATG6 function in autophagy and are implicated in powdery mildew immunity
Source: BMC Plant Biol. 2015 Apr 1;15:95. doi: 10.1186/s12870-015-0472-y (PMC4393579; doi:10.1186/s12870-015-0472-y)
Supplement: Additional file 1: Figure S1. — Sequence comparison and phylogenetic relationship of eukaryotic autophagy-related ATG6 proteins. (A) Sequence comparison between wheat ATG6 proteins and their homologs. The predicted amino acid sequences of wheat TaATG6a, 6b and 6c were aligned with homologous ATG6s from Arabidopsis (AtATG6, AAK62668), yeast (ScATG6, Q02948) and human (HsATG6, Q14457). The alignment was generated in ClustalX 2.1 and viewed in the GeneDoc 2.7 program. Numbers on the right indicate amino acid residue positions. Similarity was coded as follows: 100%, black; 80 to 100%, dark gray; 60 to 80%, light gray; and < 60%, white. The ATG6 domain (Pfam PF04111) region is under the straight lines. (B) Phylogenetic relationship of plant ATG6s. The phylogenetic tree was generated with MEGA 5 using the neighbor-joining method. The reliability of internal branches was assessed by bootstrapping, with 1000 bootstrap replicates, and the values are shown in percentages, with a branching cut-off at 50%. Wheat ATG6s are indicated by diamonds. Figure S2. Diagram of the exon-intron structures of wheat ATG6 genomic ORF sequences. Exons (boxes) and introns (lines between boxes) are drawn to scale, except introns indicated by question marks, which represent there are gaps between available genomic sequences. Two vertical short lines in intron 3 of TaATG6b represent direct short sequence repeats (TAGACTTAAATCATACTCC) delimiting the non-LTR retrotransposon (RT) sequence. [file 12870_2015_472_MOESM1_ESM.doc]

Figure S1

A

B


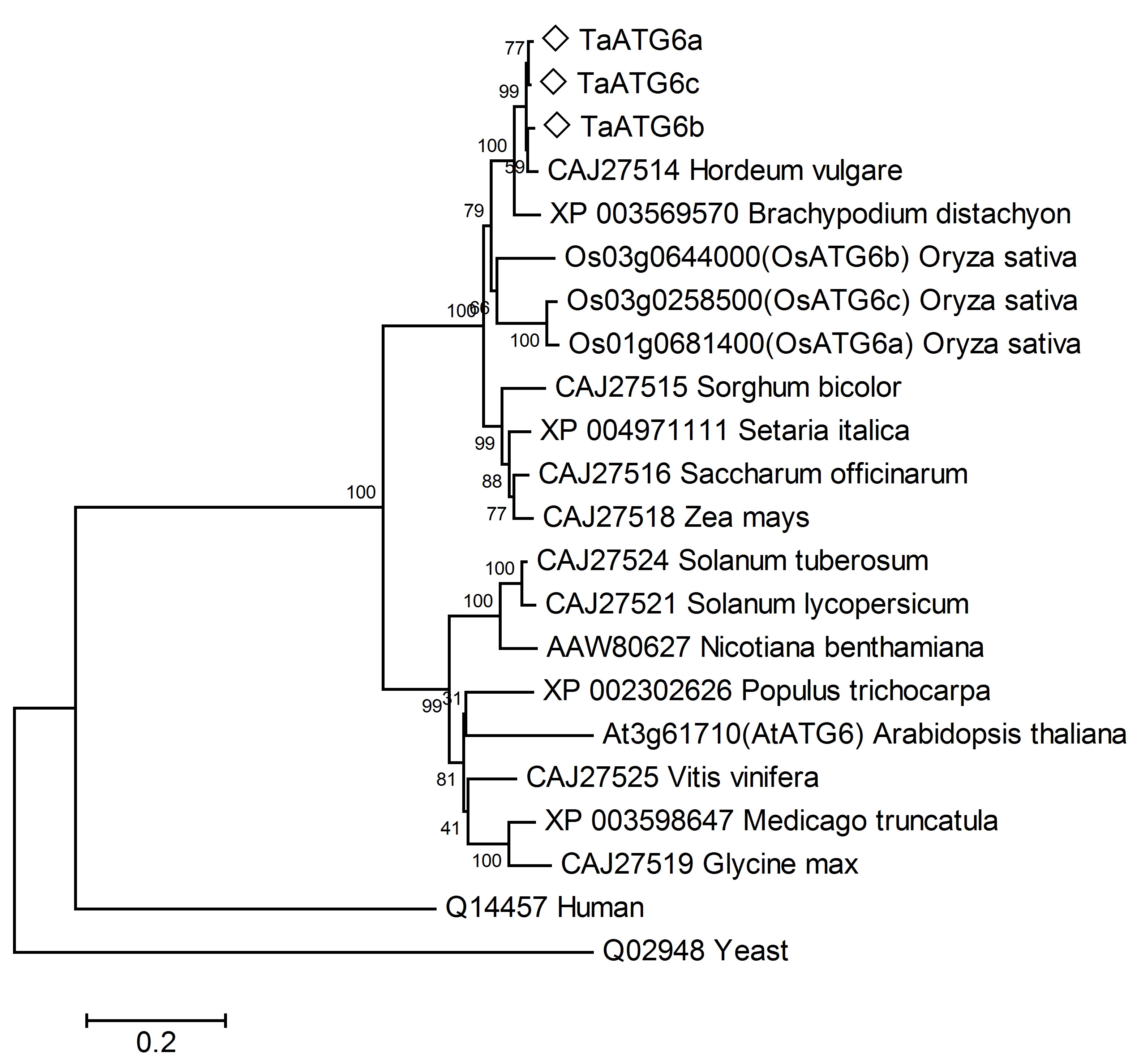


**Figure S1.** Sequence comparison and phylogenetic relationship of eukaryotic autophagy- related ATG6 proteins. **(A)** Sequence comparison between wheat ATG6 proteins and their homologues. The predicted amino acid sequences of wheat TaATG6a, 6b and 6c were aligned with homologous ATG6s from *Arabidopsis* (AtATG6, AAK62668), yeast (ScATG6, Q02948) and human (HsATG6, Q14457). The alignment was generated in ClustalX 2.1 and viewed in the GeneDoc 2.7 program. Numbers on the right indicate amino acid residue positions. Similarity was coded as follows: 100%, black; 80 to 100%, dark gray; 60 to 80%, light gray; and < 60%, white. The ATG6 domain (Pfam PF04111) region is under the straight lines. **(B)** Phylogenetic relationship of plant ATG6s. The phylogenetic tree was generated with MEGA 5 using the neighbor-joining method. The reliability of internal branches was assessed by bootstrapping, with 1000 bootstrap replicates, and the values are shown in percentages, with a branching cut-off at 50%. Wheat ATG6s are indicated by diamonds


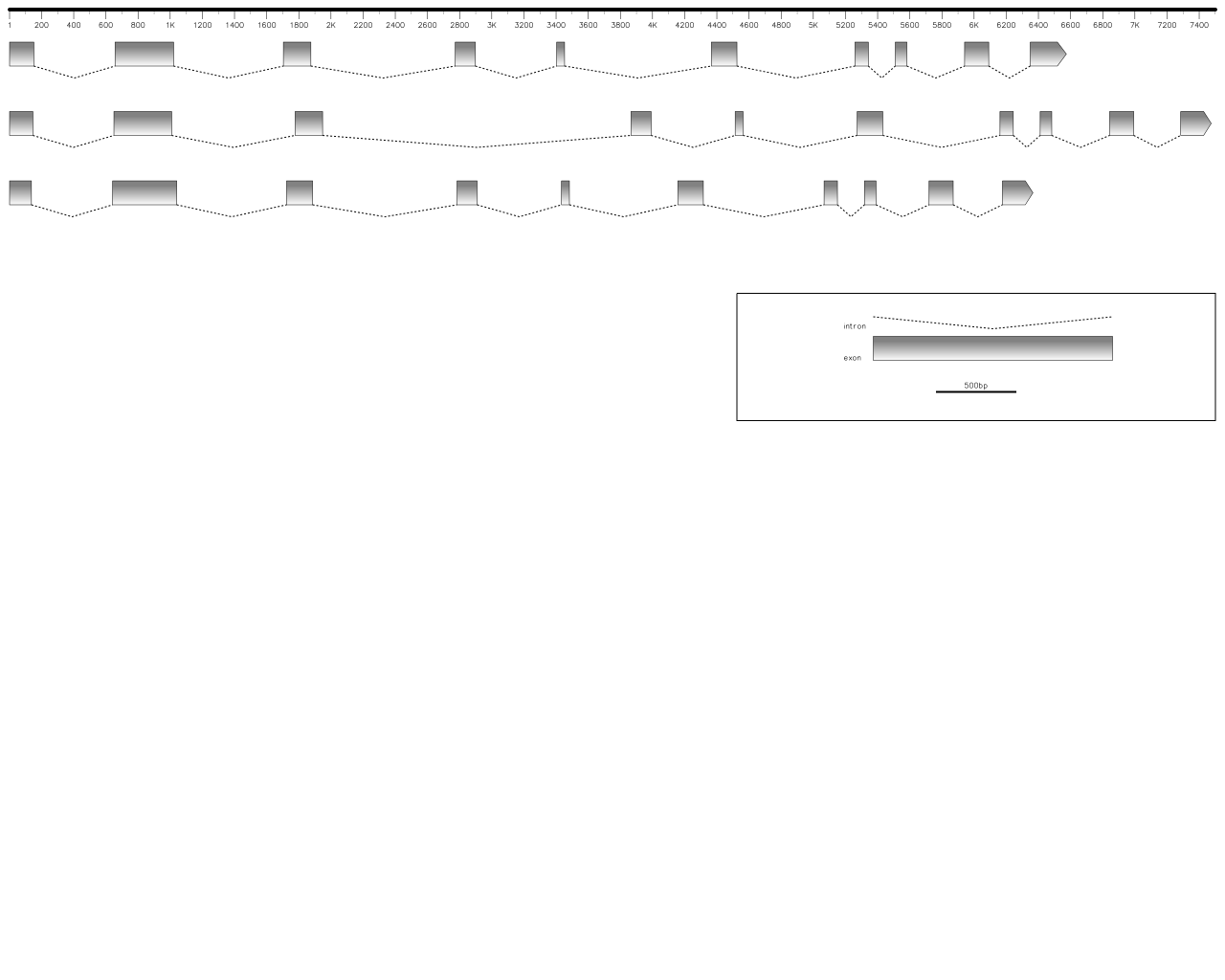


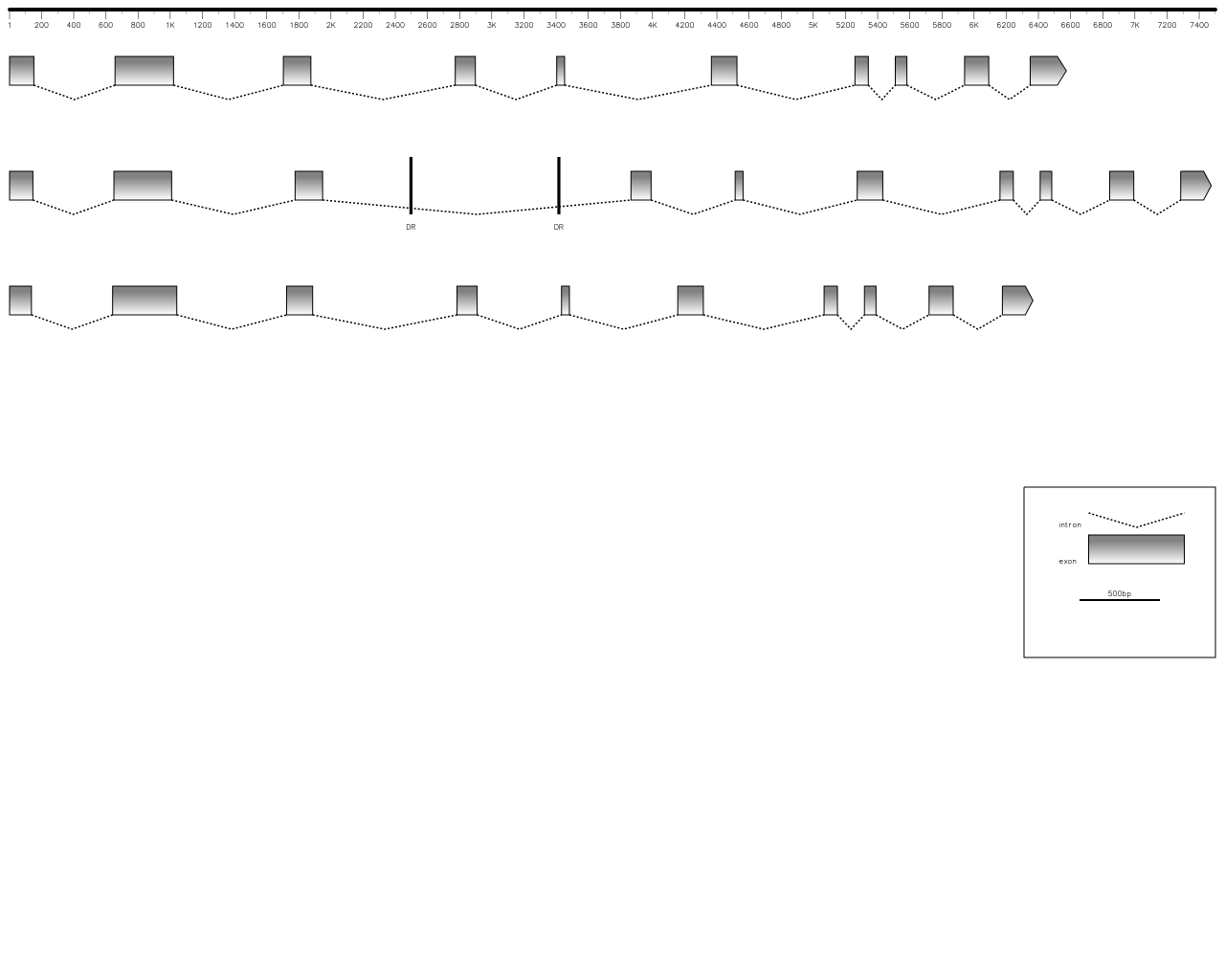


？

？

？

？

*TaATG6a*

*TaATG6b*

*TaATG6c*

504 aa

500 aa

503 aa

RT

500 bp

Figure S2

**Figure S2.** Diagram of the exon-intron structures of wheat *ATG6* genomic ORF sequences. Exons (boxes) and introns (lines between boxes) are drawn to scale, except introns indicated by question marks, which represent there are gaps between available genomic sequences. Two vertical short lines in intron 3 of *TaATG6b* represent direct short sequence repeats (TAGACTTAAATCATACTCC) delimiting the non-LTR retrotransposon (RT) sequence.
