# Supplementary material for: Wheat homologs of yeast ATG6 function in autophagy and are implicated in powdery mildew immunity
Source: BMC Plant Biol. 2015 Apr 1;15:95. doi: 10.1186/s12870-015-0472-y (PMC4393579; doi:10.1186/s12870-015-0472-y)
Supplement: Additional file 2: Table S1. — List of primers used in this paper. [file 12870_2015_472_MOESM2_ESM.doc]

Table S1 List of primers used in this paper

| **Primer name** | **Sequence（5' to 3'）** | **Product size (bp)** | **Application** |
| --- | --- | --- | --- |
| A6aF | ATGAAGCCCAAGGTTGCCGC | 1550 | Amplification of *TaATG6a* full-length cDNA by RT-PCR |
| A6aR | GAGGTCAGTGATTCCGTCTGCA |
| A6b/cF | GTTACTGCTGCGACATGAAGCC | 1517(TaATG6b)  1526(TaATG6c） | Amplification of *TaATG6b*, *6c* full-length cDNA by RT-PCR |
| A6b/cR | TCAACCCTTCTTGTTTGGAGACTG |
| NotI-A6aF | ATAAGAATGCGGCCGCATGAAGCCCAAGGTTGCCGC | 1515 | Construction of *TaATG6a* yeast expression vector |
| NotI-A6aR | ATAAGAATGCGGCCGCTCAACCCTTCTTGTTTGGAG |
| NotI-A6bF | ATAAGAATGCGGCCGCATGAAGCCCAAGGCCGCCGC | 1503 | Construction of *TaATG6b* yeast expression vector |
| NotI-A6bR | ATAAGAATGCGGCCGCTCAACCCTTCTTGTTTGGAG |
| NotI-A6cF | ATAAGAATGCGGCCGCATGAAGCCCAAGGTTGCCGC | 1512 | Construction of *TaATG6c* yeast expression vector |
| NotI-A6cR | ATAAGAATGCGGCCGCTCAACCCTTCTTGTTTGGAG |
| XhoI-A6a-GFP-F | CCGCTCGAGATGAAGCCCAAGGTTGCC | 1514 | Construction of TaATG6a-GFP expression vector |
| SpeI-A6a-GFP-R | CGGACTAGTCCACCCTTCTTGTTTGGAGA |
| XhoI-A6b-GFP-F | CCGCTCGAGATGAAGCCCAAGGCCGCC | 1502 | Construction of TaATG6b-GFP expression vector |
| SpeI-A6b-GFP-R | CGGACTAGTCCACCCTTCTTGTTTGGAGA |
| XhoI-A6c-GFP-F | CCGCTCGAGATGAAGCCCAAGGTTGCC | 1511 | Construction of TaATG6c-GFP expression vector |
| SpeI-A6c-GFP-R | CGGACTAGTCCACCCTTCTTGTTTGGAGA |
| qA6aF | CCAGGAAGAAAGAGATGCGGT | 121 | Quantitative RT-PCR of *TaATG6a* transcripts, chromosomal localization of *TaATG6a* by PCR |
| qA6aR | TCCAATCACTCCATCGTGCG |
| qA6bF | CTGGAGCACCCGATTTGACA | 116 | Quantitative RT-PCR of *TaATG6b* transcripts, chromosomal localization of *TaATG6b* by PCR |
| qA6bR | TCAATGACTTGTCAGCCGGA |
| qA6cF | ATGGTTCCTCACTTGCCTGC | 239 | Quantitative RT-PCR of *TaATG6c* transcripts, chromosomal localization of *TaATG6c* by PCR |
| qA6cR | GCGCGAAACTTGTATTGCCA |
| qA6a/b/cF | GGGATGAGATAAATGCTG | 250 | Quantitative RT-PCR of *TaATG6a*,*6b* and *6c* transcripts |
| qA6a/b/cR | TCCAAACTTATGGCAAAC |
| qTubulin-F | GTGGAACTGGCTCTGGC | 234 | Quantitative RT-PCR of the wheat β-tubulin gene transcripts |
| qTubulin-R | CGCTCAATGTCAAGGGA |
| vA6-uF | TCAGCTAGCCGGTTCAAGTGCCAGGAATG | 241 | Amplification of an upstream fragment of *TaATG6a* cDNA for VIGS vector construction |
| vA6-uR | TCAGCTAGCATTGGTTAGGCTCAACATGC |
| vA6-mF | TCAGCTAGCTTTGGAGCAGGAATCCTACA | 200 | Amplification of an middle fragment of *TaATG6a* cDNA for VIGS vector construction |
| vA6-mR | TCAGCTAGCATTCATGCCAATACCGCTCT |
| vA6-dF | CTAGCTAGCCCGGTTTGACAAAGCCATGA | 224 | Amplification of an downstream fragment of *TaATG6a* cDNA for VIGS vector construction |
| vA6-dR | CTAGCTAGCCCCACTTCAGGTTGCAAAGCATA |
